# Characterization in Potent Modulation on Voltage-Gated Na^+^ Current Exerted by Deltamethrin, a Pyrethroid Insecticide

**DOI:** 10.3390/ijms232314733

**Published:** 2022-11-25

**Authors:** Mao-Hsun Lin, Jen-Feng Lin, Meng-Cheng Yu, Sheng-Nan Wu, Chao-Liang Wu, Hsin-Yen Cho

**Affiliations:** 1 Division of Neurology, Department of Internal Medicine, Ditmanson Medical Foundation Chiayi Christian Hospital, Chiayi City 600, Taiwan; 2 Department of Emergency Medicine, Ditmanson Medical Foundation Chiayi Christian Hospital, Chiayi City 600, Taiwan; 3 Department of Physiology, National Cheng Kung University Medical College, Tainan 701, Taiwan; 4 Institute of Basic Medical Sciences, National Cheng Kung University Medical College, Tainan 701, Taiwan; 5 Department of Post-Baccalaureate Medicine, National Sun Yat-Sen University, Kaohsiung 804, Taiwan; 6 Ditmanson Medical Foundation Chiayi Christian Hospital, Chiayi City 600, Taiwan

**Keywords:** pyrethroids, voltage-gated Na^+^ current, late Na^+^ current, transient Na^+^ current, persistent Na^+^ current

## Abstract

Deltamethrin (DLT) is a type-II pyrethroid ester insecticide used in agricultural and domestic applications as well as in public health. However, transmembrane ionic channels perturbed by this compound remain largely unclear, although the agent is thought to alter the gating characteristics of voltage-gated Na^+^ (Na_V_) channel current. In this study, we reappraised whether and how it and other related compounds can make any further modifications on voltage-gated Na^+^ current (*I*_Na_) in pituitary tumor (GH_3_) cells. Cell exposure to DLT produced a differential and dose-dependent stimulation of peak (transient, *I*_Na(T)_) or sustained (late, *I*_Na(L)_) *I*_Na_; consequently, the EC_50_ value required for DLT-stimulated *I*_Na(T)_ or *I*_Na(L)_ was determined to be 11.2 or 2.5 μM, respectively. However, neither the fast nor slow component in the inactivation time constant of *I*_Na(T)_ activated by short depolarizing pulse was changed with the DLT presence; conversely, tefluthrin (Tef), a type-I pyrethroid insecticide, can accentuate *I*_Na_ with a slowing in inactivation time course of the current. The *I*_Na(L)_ augmented by DLT was attenuated by further application of either dapagliflozin (Dapa) or amiloride, but not by chlorotoxin. During pulse train (PT) stimulation, with the Tef or DLT presence, the cumulative inhibition of *I*_Na(T)_ became slowed; moreover, following PT stimuli, a large tail current with a slowly recovering process was observed. Alternatively, during rapid depolarizing pulse, the amplitude of *I*_Na(L)_ and tail *I*_Na_ (*I*_Na(Tail)_) for each depolarizing pulse became progressively increased by adding DLT, not by Tef. The recovery time constant following PT stimulation with continued presence of Tef or DLT was shortened by further addition of Dapa. The voltage-dependent hysteresis (Hys_(V)_) of persistent *I*_Na_ was differentially augmented by Tef or DLT. Taken together, the magnitude, gating, frequency dependence, as well as Hys_(V)_ behavior of *I*_Na_ exerted by the presence of DLT or Tef might exert a synergistic impact on varying functional activities of excitable cells in culture or in vivo.

## 1. Introduction

Deltamethrin (DLT, decamethrin) is a cyclopropanecarboxylate ester obtained by formal condensation between 3-(2,2-dibromovinyl)-2,2-dimethylcyclopanecarboxylic acid and cyano(3-phenoxyphenyl)methanol [1,2]. It is viewed to be the active insecticide of the proinsecticide tralomethrin [3,4,5]. Pyrethroids like DLT or tefluthrin (Tef) have been demonstrated to modify the gating characteristics of voltage-gated Na^+^ (Na_V_) channels [6,7,8,9,10]. Deltamethrin (DLT), a neurotoxic type-II pyrethroid ester insecticide [3,4,5], has been demonstrated previously to cause a reversible sequence of motor symptoms in rats involving hind limb rigidity and choreoathetosis [2,11,12,13]. Alternatively, DLT was also reported to decrease Cl^−^ currents through voltage-dependent Cl^−^ channels and this action probably contributes the most to the features of poisoning with DLT or other type II pyrethroids [14,15,16]. At relatively high concentrations, pyrethroids can also act on GABA-gated Cl^−^ channels, which may be responsible for the seizures seen with severe type-II poisoning [2,17].

It has been established that nine isoforms (i.e., Na_V_1.1–1.9 [or SCN1A–SCN5A and SCN8A–SCN11A]) of the voltage-gated Na^+^ (Na_V_) channels are widely distributed in mammalian excitable tissues, which include the central or peripheral nervous system, and the endocrine or neuroendocrine system [18,19,20]. The eukaryotic versions of these Na_V_-channel proteins are comprised of a single subunit which contains four six-transmembrane pseudodomains [20,21]. Upon rapid depolarization, the Na_V_ channels, by which macroscopic voltage-gated Na^+^ currents (*I*_Na_) are encoded, are characterized by going through rapid transitions from the closed (resting) state to the open state, and then by swiftly changing to the inactivated state [20,21]. The inactivation of *I*_Na_ has been also demonstrated to accumulate before being stimulated during repetitive short depolarizing pulses [22,23,24,25]. Consequently, once being evoked, the increased magnitude of *I*_Na_ can quickly depolarize the cell membrane through positive feedback cycle and, in turn, elicit the upstroke of the action potentials, thereby intrinsically governing the amplitude, frequency, and/or pattern of firing action potentials, as well as hormonal secretion, in an array of excitable cells [20,21,26,27]. On the other hand, the aberrant changes in Na_V_ (i.e., Na_V_1.2) channel activity occurring in corticostriatal circuits of adult mice were also reported to elevate neuronal excitability [28].

Like the action of tefluthrin (Tef) [7,8,16,26,29], deltamethrin (DLT) was used to kill a wide range of insects [2,6,30]. There is a growing concern over human or animal poisoning as aberrant use in these esters. However, whether and how deltamethrin (DLT) or other structurally similar pyrethroids (e.g., Tef) is able to modify the magnitude, gating kinetics, frequency dependence, and/or voltage-dependent hysteresis (Hys_(V)_) of *I*_Na_ remains mostly obscure, although they are recognized to augment the *I*_Na_ magnitude [6,7,8,9].

In light of the aforementioned considerations, we wanted to extensively explore the electrophysiological effects of DLT and other related compounds (e.g., Tef) in pituitary GH_3_ sommatolactotrophs with either a single voltage-clamp pulse or pulse train (PT) stimulation. The tetrodotoxin (TTX)-sensitive *I*_Na_, which is responsible for the generation of action potentials, has been identified in pituitary tumor (GH_3_) cells [18]. The GH_3_ cell line has been demonstrated previously to express the α-subunits of Na_V_1.1, Na_V_1.2, Na_V_1.3, and Na_V_1.6, as well as the β1 and β3-subunits of Na_V_ channel [18,31]. In the current investigations, we intended to (1) evaluate if DLT has any perturbations on the peak (transient, *I*_Na(T)_) and sustained (late, *I*_Na(L)_) components of *I*_Na_ intrinsically in these cells; (2) examine if this compound affects either magnitude or time course of *I*_Na_ during as well as following 1-s pulse train (PT) stimulation; (3) explore whether or not the Hys_(V)_’s behavior of persistent *I*_Na_ (*I*_Na(P)_) could be seriously disturbed by the presence of DLT; and (4) the molecular docking between the DLT molecule and the hNa_V_1.5 channel was also predicted. The present results disclosed that the differential and dose-dependent stimulation of *I*_Na(T)_ and *I*_Na(L)_ by DLT as well as its perturbations either on *I*_Na_ occurring during or following PT stimulation, or on Hys_(V)_ properties of *I*_Na(P)_ may potentially converge to engage in a great impact on electrical behaviors of mammalian excitable cells (e.g., GH_3_ cells).

## 2. Results

### 2.1. Modification by Deltamethrin (DLT) or Tefluthrin (Tef) on Voltage-Gated Na^+^ Current (I_Na_) Measured from Pituitary GH_3_ Cells

**Figure 1:**
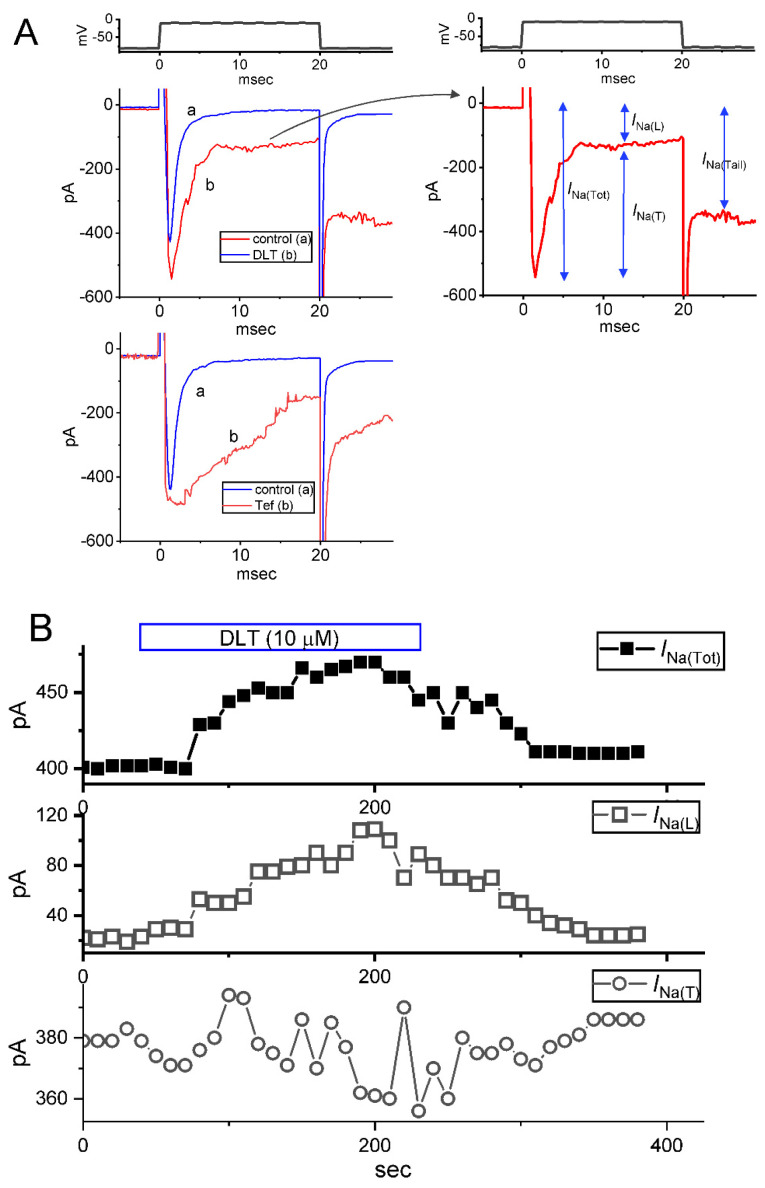

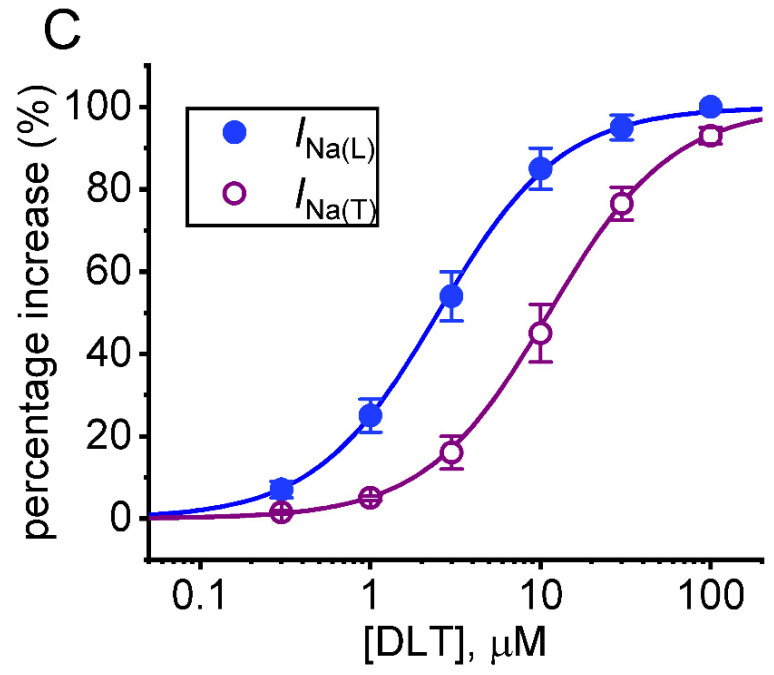
Effect of deltamethrin (DLT) or tefluthrin (Tef) on voltage-gated Na^+^ current (*I*_Na_) measured from pituitary GH_3_ lactotrophs. This set of experiments was made in cells placed in Ca^2+^-free, Tyrode’s solution containing 10 mM tetraethylammonium chloride (TEA), and the measuring electrode was filled with an internal solution enriched with Cs^+^. (**A**) Exemplar current traces obtained in (a, blue color) the control conditions (i.e., neither DLT nor Tef was present) and during cell exposure to either 10 μM DLT (b, upper, red color) or 10 μM Tef (b, lower, red color). The voltage-clamp protocol is illustrated atop recorded current traces. The graph shown in the right side of (**A**) denotes the expanded record from the observed current trace (red color) in the presence of 10 μM DLT and the definition of transient Na^+^ current (*I*_Na(T)_), late Na^+^ current (*I*_Na(L)_), total Na^+^ current (*I*_Na(Tot)_), or tail Na^+^ current (*I*_Na(Tail)_) is marked (indicated with blue double arrows). (**B**) Time course of effects of 10 μM DLT on the amplitude of *I*_Na(Tot)_ (upper), *I*_Na(L)_ (middle), and *I_Na(T)_* (lower). Each point was taken at a rate of 0.1 Hz. The horizontal bar shown above indicated the application of DLT. (**C**) Concentration-dependent relationship of DLT on *I*_Na(T)_ (purple open circles) or *I*_Na(L)_ (blue solid circles) activated by short depolarizing step. Each data point in this graph represents mean ± SEM of 9 cells. According to the averaged data, the smooth line represents the best fit to the Hill equation as described in Materials and Methods.

In the first stage of whole-cell current recordings, we measured the effects of DLT or Tef on the magnitude and inactivation time course of *I*_Na_ activated in response to abrupt depolarizing pulse. We placed cells in Ca^2+^-free, Tyrode’s solution containing 10 mM tetraethylammonium chloride (TEA), and the pipette used was filled up with a Cs^+^-containing solution. As demonstrated in Figure 1A, two minutes after cells were continually exposed to DLT or Tef at a concentration of 10 μM, the amplitude in the transient (*I*_Na(T)_) or late (*I*_Na(L)_) component of *I*_Na_ activated by 20-ms depolarizing pulse from −80 to −10 mV was progressive raised. For example, as the rectangular voltage step from −80 to −10 mV with a duration of 20 ms was given (indicated in the uppermost part of Figure 1A) to activate *I*_Na_, the addition of 10 μM DLT was found to result in a striking increase in either *I*_Na(T)_ or *I*_Na(L)_ amplitude to 512 ± 17 pA (*n* = 9, *p* < 0.05) or 128 ± 9 pA (*n* = 9, *p* < 0.05) from control values of 401 ± 15 or 22 ± 5 pA (*n* = 9), respectively. After washout of DLT, *I*_Na(T)_ or *I*_Na(L)_ was returned to 409 ± 17 or 28 ± 7 pA (*n* = 9). Likewise, the presence of 10 μM Tef also measurably increased *I*_Na(T)_ or *I*_Na(L)_ amplitude from 409 ± 14 pA (*n* = 9) or 32 ± 6 pA (*n* = 9) to 499 ± 16 pA (*n* = 9, *p* < 0.05) or 162 ± 11 pA (*n* = 9, *p* < 0.05), respectively. However, with cell exposure to 10 μM DLT, neither fast nor slow time constant of *I*_Na(T)_ inactivation in response to rapid membrane depolarization was evidently changed. Alternatively, with the presence of 10 μM Tef, the slow component in the time constant of *I*_Na(T)_ inactivation was strikingly raised to 19 ± 2 msec (*n* = 9, *p* < 0.05) from a control value of 0.9 ± 0.2 msec (*n* = 9). The time course of effects of DLT (10 μM) on the amplitude of *I*_Na(Tot),_ *I*_Na(L)_ or *I*_Na(T)_ is illustrated in Figure 1B. Of note, during exposure to 10 mM DLT, the amplitude of *I*_Na(Tot)_, *I*_Na(L)_, or *I*_Na(T)_ was increased to 177 ± 21 % (*n* = 8), 1752 ± 105 % (i.e., around 1.7-fold) (*n* = 8), or 24 ± 6% (*n* = 8), respectively.

Figure 1C demonstrates that the addition of DLT to the bath can concentration-dependently increase the amplitude of *I*_Na(T)_ or *I*_Na(L)_ activated by short depolarizing step. According to the Hill equation stated under Materials and Methods, the EC_50_ value needed for DLT-stimulated *I*_Na(T)_ or *I*_Na(L)_ observed in GH_3_ cells was calculated as 11.2 or 2.5 µM, respectively. Consistent with previous studies [6,8], the experimental observations, therefore, enable us to reflect that the DLT presence exerts a stimulatory action on the magnitude of *I*_Na(T)_ and *I*_Na(L)_ natively expressed in GH_3_ cells, and that this compound tends to be selective for *I*_Na(L)_ over *I*_Na(T)_ during rectangular depolarizing pulse.

### 2.2. Comparison among Effects of Tef, DLT, Tef plus Chlorotoxin (ChloroTx), DLT plus ChloroTx, Tef plus Dapaglifozin (Dapa), DLT plus Dapa, and DLT plus Amiloride on I_Na(L)_ Amplitude Measured from GH_3_ Cells

**Figure 2:**
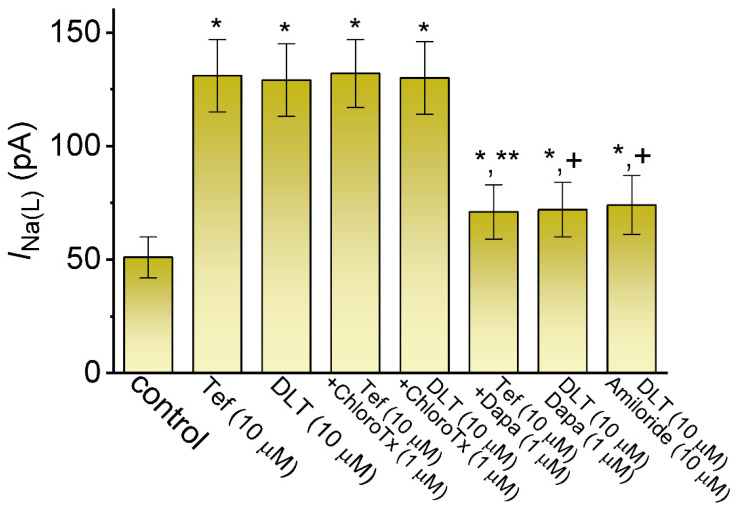
Comparison among effects of Tef, DLT, Tef plus chlorotoxin (ChloroTx), DLT plus ChloroTx, Tef plus dapagliflozin (Dapa), DLT plus Dapa, and DLT plus amiloride on the amplitude of *I*_Na(L)_ measured from GH_3_ cells. The *I*_Na_ was elicited by 20 ms depolarizing voltage command from −80 to −10 mV for a duration of 20 ms at a rate of 0.2 Hz. The *I*_Na(L)_ amplitudes during exposure to different tested compounds were measured at the end of each depolarizing step. Each bar represents the mean ± SEM (*n* = 8). ^*^ Significantly different from control (*p* < 0.05), ** significantly different from Tef (10 μM) alone group (*p* < 0.05), and ^+^ significant different from DLT (10 μM) alone group (*p* < 0.05).

Exposure to pyrethroids (e.g., DLT) has been previously demonstrated to activate Cl^−^ currents [14,15,16]. We further compared the effects of Tef, DLT or their combinations with ChloroTx, Dapa, or amiloride on *I*_Na(L)_ amplitude. ChloroTx was reported to suppress Cl^−^ current, Dapa was an inhibitor of *I*_Na(L)_ [32,33], and amiloride can attenuate the pyrethroids-stimulated sodium transport [16]. As summarized in Figure 2, with continued presence of Tef or DLT, the further exposure to ChloroTx (1 μM) failed to modify their stimulation of *I*_Na(L)_. Dapa (10 mM) or amiloride (10 mM) alone decrease the *I*_Na(L)_ amptitude to 31 ± 2 pA (*n* = 8, *p* < 0.05) or 28 ± 2 pA (*n* = 8, *p* < 0.05) from control value of 50.3 ± 3 pA (*n* = 8). Moreover, the subsequent presence of either Dapa (10 μM) or amiloride (10 μM) was able to attenuate Tef- or DLT-mediated increase of *I*_Na(L)_ effectively. The results prompted us to suggest that either Dapa or amiloride could directly cause an inhibitory effect on the amplitude of *I*_Na(L)_ observed in GH_3_ cells [32,33].

### 2.3. Effect of DLT on Mean Current Versus Voltage (I-V) Relationship of I_Na(T)_ and I_Na(L)_

**Figure 3:**
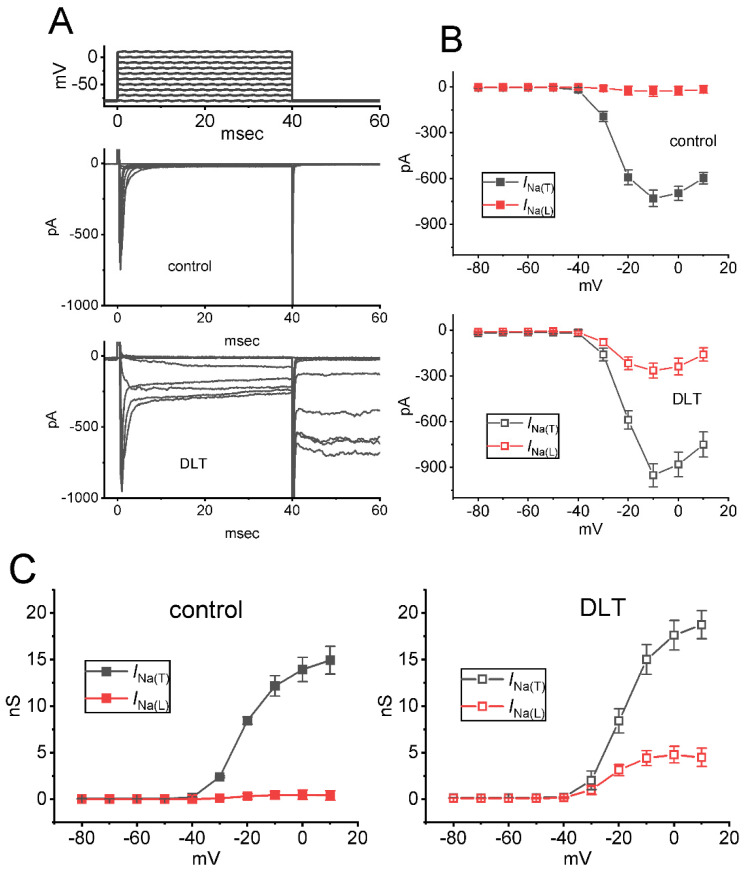
Effect of DLT on the steady-state current versus voltage (I-V) relationship of I_Na(T)_ and I_Na(L)_ identified from GH_3_ cells. In this set of experiments, we held each cell at −80 mV, and varying depolarizing command voltages from −80 to +10 mV in 10-mV steps were delivered to evoke I_Na(T)_ and I_Na(L)_. (**A**) Exemplar current traces obtained either in the control condition (upper) or with the presence of 10 μM DLT (lower). The uppermost part is the voltage-clamp protocol given. (**B**) The mean I-V relationship of I_Na(T)_ (black symbols) or I_Na(L)_ (red symbols) in control (upper, solid symbols) and during exposure to 10 μM DLT (lower, open symbols) (mean ± SEM; *n* = 8 for each point). Either I_Na(T)_ or I_Na(L)_ was measured at the beginning or end of each depolarizing pulse. (**C**) Conductance versus voltage relationship of I_Na(T)_ (black symbols) or I_Na(L)_ (red symbols) in the control period (left side) and during cell exposure to 10 μM DLT (right side) (mean ± SEM; *n* = 8 for each point).

We next explored any perturbations of this compound on the amplitude of *I*_Na(T)_ or *I*_Na(L)_ measured from the different level of membrane potentials. As demonstrated in Figure 3A,B, a steady-state *I-V* relationship of I_Na(T)_ and I_Na(L)_ acquired with or without the DLT (10 μM) presence was established in these cells. The appearance of 10 μM DLT resulted in a striking increase in the I_Na(T)_ or I_Na(L)_ amplitude elicited by abrupt depolarizing steps. For example, when the tested cells were rapidly depolarized from −80 to −10 mV, the addition of 10 μM DLT raised either I_Na(T)_ or I_Na(L)_ magnitude from 729 ± 54 to 952 ± 76 pA (*n* = 8, *p* < 0.05), or from 26 ± 34 to 265 ± 48 pA (*n* = 8, *p* < 0.05), respectively. However, the steady-state *I-V* relationship of *I*_Na(T)_ or *I*_Na(L)_ remained unaffected during exposure to 10 μM DLT, despite a marked increase in I_Na(T)_ or I_Na(L)_ magnitude. The relationship (i.e., G-V relationship) for the conductance of *I*_Na(T)_ or *I*_Na(L))_ with or without the application of 10 μM DLT was also established and depicted in Figure 3C. The V_1/2_ value for G-V relationship of *I*_Na(T)_ or *I*_Na(L)_ between the absence and presence of 10 mM DLT did not differ significantly {(−19.9 ± 1.8 mV [control] versus −20.5 ± 1.7 mV [in the presence of DLT]; *n* = 8, *p* > 0.05, for the results of *I*_Na(T)_), or (−18.4 ± 1.6 mV [control] versus −18.5 ± 1.6 mV [in the presence of DLT]; *n* = 8, *p* > 0.05, for the results of *I*_Na(L)_)}.

### 2.4. Tef- or DLT-Mediated Slowing in Cumulative Inhibition of I_Na(T)_ during Rapid Depolarizing Stimuli

**Figure 4:**
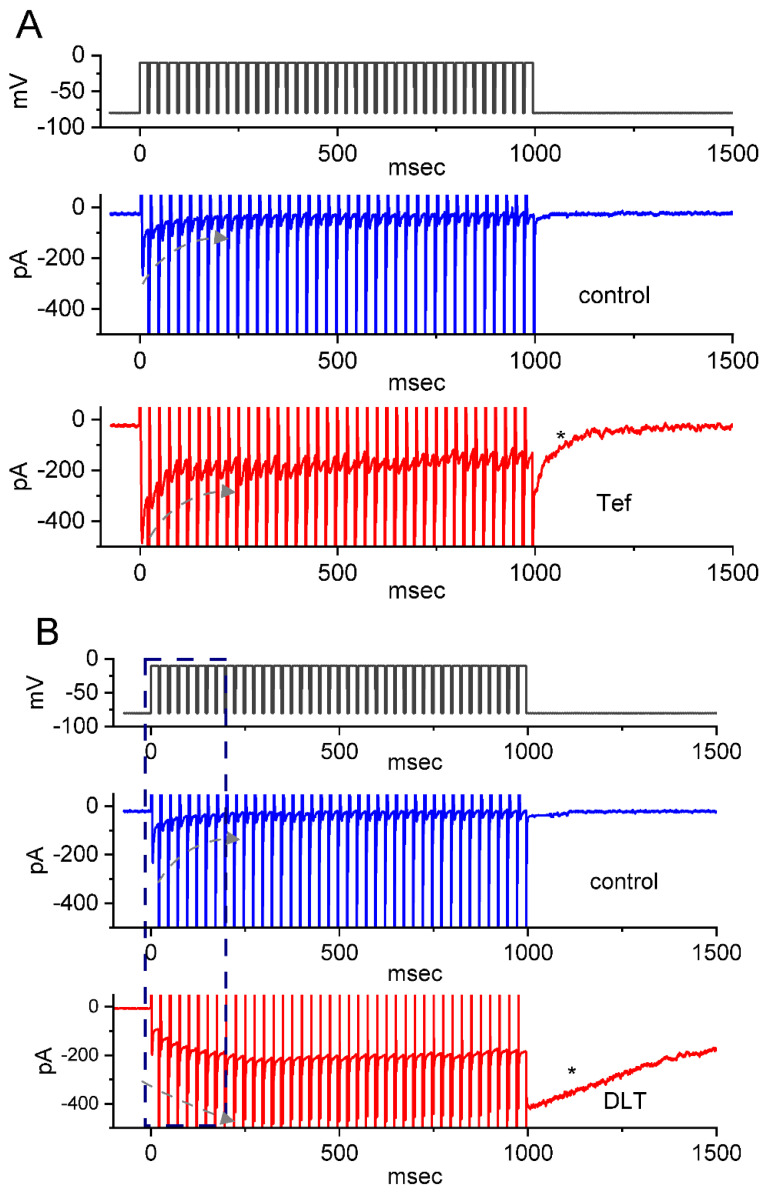

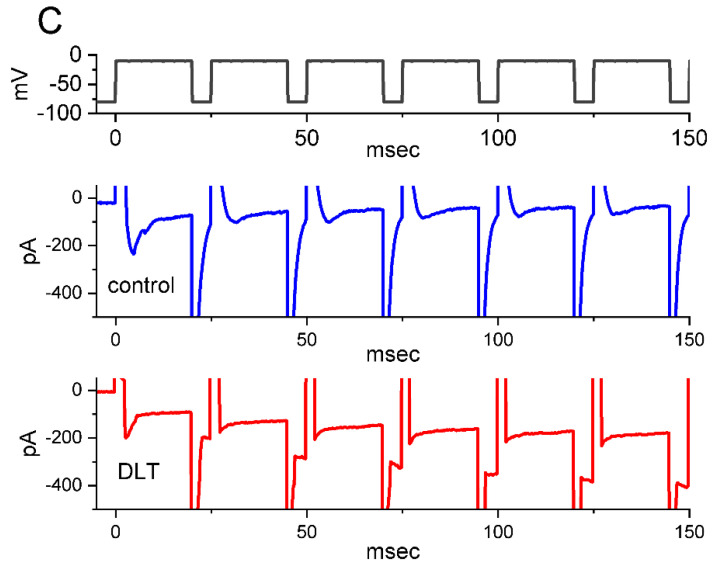
Effects of Tef (**A**) or DLT (**B**,**C**) on *I*_Na_ evoked by a train of depolarizing pulses (i.e., pulse train [PT] stimulation) in GH_3_ cells. The train given consists of 40–20 ms pulses (stepped to −10 mV) separated 5 ms intervals at −80 mV for a total duration of 1 sec. In (**A**) or (**B**), exemplar current traces acquired in the control period (i.e., neither Tef nor DLT was present, upper part, blue color) and during cell exposure to 10 μM Tef (lower part, red color) or 10 μM DLT (lower part, red color) are illustrated, respectively. The voltage-clamp protocol (black color) atop current traces in (**A**–**C**) is illustrated. The black dashed arrows in (**A**) or (**B**), respectively, indicate the direction of current changes (i.e., either decay or rise) over time in an exponential fashion, while the asterisk shows a large inward deflection following PT stimulation with cell exposure to 10 μM Tef (upper) or 10 μM DLT (lower). (**C**) Expanded records (i.e., potential or current traces) from the broken box in (**B**).

**Figure 5:**
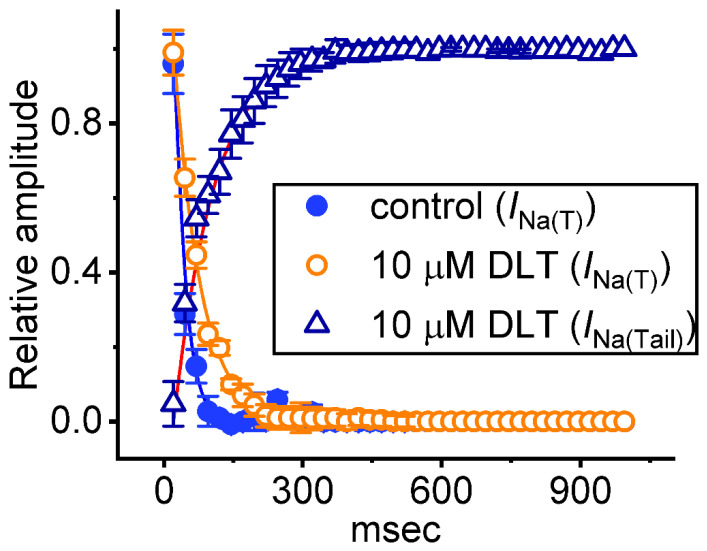
Relationship of *I*_Na(T)_ or *I*_Na(Tail)_ versus the pulse train (PT) duration in the absence (blue filled circles) and presence (orange open circles or blue open triangles) of 10 μM DLT (mean ± SEM, *n* = 8 for each point). The observed I_Na(T)_ or I_Na(L)_ was measured as indicated in the right side of Figure 1. The continuous smooth lines, over which the experimental data points are overlaid, were optimally fitted by a single exponential (i.e., exponential decrease or increase). Notably, during PT stimulation, cell exposure to DLT can increase the decaying time constant of I_Na(T)_ inactivation; however, it led to a progressive increase (i.e., staircase increase) in the amplitude of I_Na(Tail)_.

It has been demonstrated that, prior to being activated during repetitive short pulses, the inactivation of *I*_Na(T)_ is able to accumulate [22,23,25,34,35,36]. For this reason, we next explored if Tef or DLT could modify the extent of *I*_Na(T)_ activated either during or following the PT depolarizing stimuli. In this set of measurement, the stimulus protocol, consisting of repetitive depolarization of −10 mV (20 ms in each pulse with a rate of 40 Hz for 1 s), was applied to the tested cells which were voltage-clamped at −80 mV. In accordance with earlier reports [22,23,24,25,36], as demonstrated in Figure 4 and Figure 5, during the control period (i.e., neither Tef nor DLT was present), the exponential time course of *I*_Na(T)_ inactivation observed in GH_3_ cells was observed during a 1-s repetitive depolarization from −80 to −10 mV, and an evolving decaying time constant of 22.1 ± 2.8 ms (*n* = 8) was then yielded. In other words, there appeared to be a progressive current decay (indicated with the dashed arrows in Figure 4A) with a single-exponential process. It also needs to be noted that with cell exposure to DLT (10 μM), the time constant of *I*_Na(T)_ decaying activated during the same train of depolarizing pulses was increased to 56.4 ± 3.9 ms (*n* = 8), apart from a progressive increase in *I*_Na(L)_ or *I*_Na(Tail)_ (i.e., appearance of tail current following 1-s PT stimulation) magnitude. Of additional notice, a significant increase in tail *I*_Na_ (*I*_Na(Tail)_) (blue open triangles in Figure 5) with a rising time constant of 87.4 ± 4.6 ms (*n* = 8) was found in the presence of 10 μM DLT (Figure 4B,C and Figure 5); however, cell exposure to 10 μM Tef resulted in a gradual decay in *I*_Na(L)_ with a decaying time constant of 26.3 ± 2.7 ms (*n* = 8). Alternatively, with continued exposure to 10 mM DLT, further addition of Dapa (10 mM) or Ami (10 mM) significantly decreased the time constant of *I*_Na(L)_ during PT stimulation. Table 1 summarizes the results showing effects of DLT, DLT plus dapagliflozin (Dapa), DLT plus amiloride (Ami) on either the decaying time constant of *I*_Na(T)_ during PT stimulation or the rising time constant of *I*_Na(L)_ during the same PT stimulation, as well as the time constant of *I*_Na(Tail)_ recovery evoked following PT stimulation.

**Figure 6:**
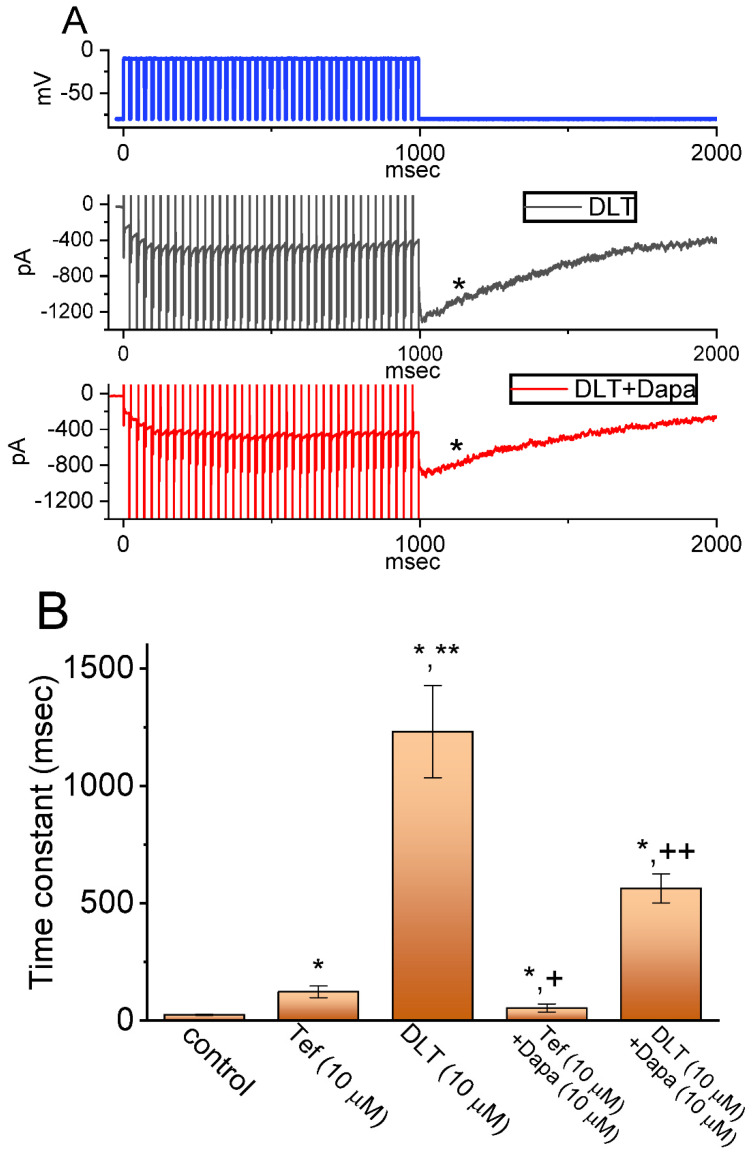
Effect of DLT or DLT plus Dapa on *I*_Na_ evoked by PT stimulation identified from GH_3_ cells. The PT stimulation was applied in exactly the same way as utilized in Figure 4. (**A**) Exemplar current traces obtained in the presence of DLT (10 μM) alone (black color) or DLT (10 μM) plus Dapa (10 μM) (red color). The upper part shows the voltage-clamp protocol (blue color) given, whereas asterisk denotes the emergence of the current recovery immediately following PT stimulation. (**B**) Summary bar graph demonstrating effects of Tef, DLT, Tef plus Dapa, and DLT plus Dapa on the recovery time constant of I_Na_ following PT stimulation (mean ± SEM; *n* = 8 for each bar). * Significantly different from control (*p* < 0.01), ** significantly different from Tef (10 μM) alone group (*p* < 0.05), ^+^ significantly different from Tef (10 μM) alone group (*p* < 0.05), and ^++^ significantly different from DLT (10 μM) alone group (*p* < 0.05).

Moreover, with the DLT presence, an exponential increase in *I*_Na(L)_ during PT stimuli occurring over time was also observed (Figure 4B,C). Following 1-s PT stimulation, as cells were continually exposed to 10 μM DLT, there appeared to be a large inward current (i.e., *I*_Na(Tail)_) accompanied by a gradual recovery (indicated with asterisk in Figure 4B) in the second timescale with a recovery time constant of 1.23 ± 0.19 s (*n* = 8) (Figure 4B,C). The appearance of *I*_Na(Tail)_ could reflect changes in the magnitude of *I*_Na(P)_, and the *I*_Na(L)_ and *I*_Na(P)_ evoked during an extended period of time were thought to share the same Na_V_ channels [22,26]. Likewise, with the presence of 10 μM Tef, the recovery time constant of *I*_Na(Tail)_) (or *I*_Na(P)_) acquired following PT stimuli was estimated to be 123 ± 25 ms (*n* = 8), a value which is different from DLT-induced change in the recovery time constant of *I*_Na(Tail)_ following PT stimuli. In contrast, during the control period (i.e., neither Tef nor DLT was present), the recovery time constant of the current following PT stimulation was rather small (i.e., 25 ± 3 ms [*n* = 8]) (Figure 6B). Moreover, with continued presence of DL (10 mM), further addition of either Dapa (10 mM) or Ami (10 mM) significantly attenuated the recovery time constant of *I*_Na(T)_ evoked following PT stimulation, as summarized in Table 1.

Additionally, with continued exposure to Tef (10 μM) or DLT (10 μM), further addition of dapagliflozin (Dap) at a concentration of 10 μM resulted in an attenuation of the drastic appearance of large inward *I*_Na(Tail)_ following PT stimuli, as demonstrated by a respective reduction in the recovery time constant of the current to 54 ± 17 ms (*n* = 8, *p* < 0.05) or 564 ± 62 ms (*n* = 8, *p* < 0.05) estimated during further presence of Dapa (10 μM) (Figure 6B). Taken together, these results prompted us to reflect that the presence of DLT can act as a striking slowing the deactivating kinetics of *I*_Na(Tail)_ (or *I*_Na(P)_) following return to the holding potential at −80 mV. Therefore, during 1-s PT stimulation, insufficient period of time was allowed for *I*_Na_ recovery. As a result, particularly during exposure to DLT, single *I*_Na_ deactivation during PT stimulation presently given (i.e., at a rate of 40 Hz) could be apparently incomplete, thereby leading to frequency-dependent ‘accumulation’ of the Na_V_-channel activated state. Therefore, the response of Tef- and DLT-mediated *I*_Na(L)_ or *I*_Na(Tail)_ was overly distinguishable. In other words, one (i.e., the Tef presence) is progressive decay of *I*_Na(L)_ during a train of depolarizing pulses, while the other (i.e., the DLT presence) exhibits a staircase increase in *I*_Na(L)_. Moreover, upon continued exposure to 10 μM DLT, the subsequent addition of Dapa (10 μM) could measurably attenuate DLT-mediated increase I_Na(Tail)_ during PT stimuli as well as shortened the recovery time constant of *I*_Na(P)_ following repetitive depolarizing stimuli (Figure 6A,B).

### 2.5. Effect of Tef or DLT on the Strength of Voltage-Dependent Hysteresis (Hys_(V)_) of Persistent I_Na_ (I_Na(P)_) Elicited by an Upright Isosceles-Triangular Ramp Voltage (V_ramp_)

**Figure 7:**
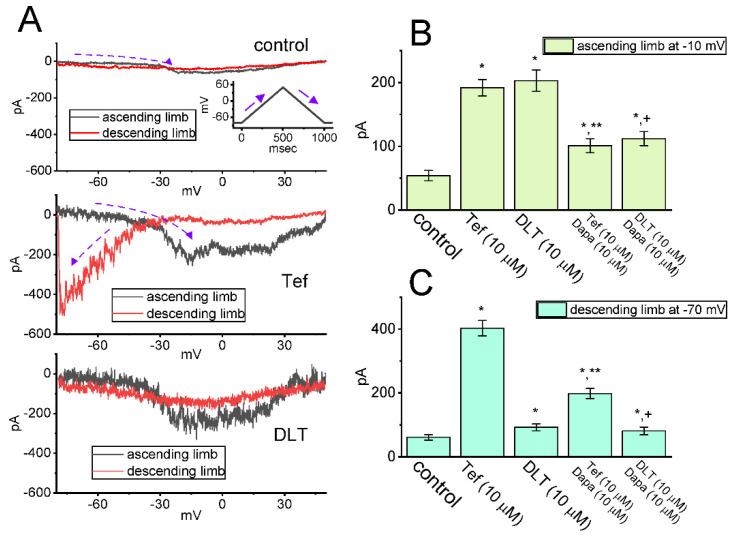
Modifications by Tef or DLT on the strength of voltage-dependent hysteresis (Hys_(V)_) in persistent *I*_Na_ (*I*_Na(P)_) present in GH_3_ cells. In this set of whole-cell current recordings, the examined cell was voltage-clamped at −80 mV and we then delivered the isosceles-triangular ramp voltage (V_ramp_) for a duration of 1 s (i.e., a ramp speed of ±0.26 mV/ms) to activate *I*_Na(P)_. (**A**) Exemplar current traces obtained in the control period (upper) and in the presence of 10 μM Tef (middle) or 10 μM DLT (lower). The ascending (upsloping) limb is indicated in black color, where the descending (downsloping) one is in the red color. Inset in the upper part of (**A**) shows the voltage-clamp protocol applied, whereas the dashed arrow indicates the direction of potential or current trajectory by which time goes. In (**B**) or (**C**), summary bar graph, respectively, demonstrates the effect of Tef (10 μM), DLT (10 μM), Tef (10 μM) plus Dapa (10 μM), and DLT (10 μM) plus Dapa (10 μM) on *I*_Na(P)_ amplitude activated by upsloping (at −10 mV) or downsloping limb (at the level of −70 mV) of double V_ramp_ (mean ± SEM; *n* = 8 for each bar). * Significantly different from control (*p* < 0.05), ** significantly different from Tef (10 μM) alone group (*p* < 0.05), and ^+^ significantly different from DLT (10 μM) alone group (*p* < 0.05).

The nonlinear Hys_(V)_ behavior residing in I_Na(P)_ has been recently disclosed with a figure-of-eight (i.e., ∞-shaped) configuration as current traces were robustly activated by an upright double V_ramp_ (i.e., ascending and descending limbs of triangular V_ramp_) [33]. In this regard, efforts were made to explore if the existence of Tef or DLT could have any different adjustments on the Hys_(V)_’s behavior elicited in response to such upright V_ramp_. This separate set of measurements was performed in GH_3_ cells which were placed in Ca^2+^-free Tyrode’s solution, and we filled up the measuring electrodes with a solution containing Cs^+^. The tested cells were maintained at −80 mV and an upright double V_ramp_ ranging between −80 and +50 mV for a duration of 1 s (i.e., ramp speed of ±0.26 mV/ms) was afterwards applied to them. As shown in Figure 7A, during cell exposure to Tef (10 μM) or DLT (10 μM), current traces in response to such triangular V_ramp_ are distinguishable, although two types of hysteretic loops (i.e., low- and high-threshold loops) became overly noticeable during the presence of Tef or DLT. In particular, the strength of low-threshold hysteretic loop during such V_ramp_ became considerably larger in the presence of Tef (10 μM), as compared with that during exposure to DLT. For example, as cells were continually exposed to Tef (10 μM), the amplitude of I_Na(P)_ at the descending limb of V_ramp_ (i.e., at the level of −70 mV) resulted in a striking increase by 6.6 folds from 61 ± 9 to 403 ± 24 pA (*n* = 8, *p* < 0.05); conversely, upon the presence of DLT (10 μM), I_Na(P)_ amplitude at the same level was increased only by 1.5 folds (Figure 7C). However, upon cell exposure to Tef (10 μM) or DLT (10 μM), the I_Na(P)_ amplitude at the ascending limb (i.e., at −10 mV) was increased to 3.6 or 3.7 folds, respectively, which did not differ significantly between these two compounds. From these data, it was plausible to assume that the strength of I_Na(P)_’s Hys_(V)_ in response to long-lasting double V_ramp_ was susceptible to being enhanced during Tef or DLT presence; moreover, the low-threshold loop of Hys_(V)_ appeared to be more sensitive to augmentation by Tef than that by DLT.

## 3. Discussion

In the current investigations together with previous studies, we provided the evidence to unveil that the presence of DLT, known to be a type II pyrethroid, was able to exert stimulatory actions on vastly different types of *I*_Na_, including *I*_Na(T)_, *I*_Na(L)_, *I*_Na(Tail)_, and *I*_Na(P)_, seen in pituitary tumor (GH_3_) cells. It is likely, therefore, that the endocrine disrupting potential caused by the existence of DLT or other structurally similar pyrethroids, as demonstrated recently [2,16,20,27,37,38,39,40,41], could be highly linked to the excitatory actions on varying types of *I*_Na_ presented herein, presuming that similar pharmacological or toxicological actions take place in variable types of endocrine or neuroendocrine cells present in vivo [2,3,7,11,13,42], although pyrethroids are thought to be around 2250 times more toxic than mammals [12,13].

Upon cell exposure to DLT, the observed *I*_Na(L)_ activated in response to short depolarizing step was noticed to be stimulated to a greater extent than the *I*_Na(T)_. The EC_50_ values required for DLT-stimulated *I*_Na(T)_ and *I*_Na(L)_ in GH_3_ cells were estimated to be 11.2 and 2.5 μM, these values which was noted to differ significantly by 4.5 folds (Figure 1). However, the further addition of chlorotoxin (ChloroTx), still in continued presence of tefluthrin (Tef) or DLT, failed to modify Tef- or DLT-stimulated *I*_Na(L)_, although either dapagliflozin (Dapa) or amiloride could effectively reverse their increase in *I*_Na(L)_ amplitude. Tef is a Type I pyrethroid insecticide [8,10,26]. The overall steady-state *I-V* relationship of *I*_Na(T)_ or *I*_Na(L)_ during exposure to DLT remained unchanged; furthermore, the inactivation time course of *I*_Na(T)_ during brief step depolarizing did not differ between the absence and presence of DLT. However, the magnitude of *I*_Na(T)_ following the PT stimulation (i.e., 40-Hz repetitive depolarizing pulse) tended to be pronouncedly larger as well as its decaying time course became slowed in the presence of Tef or DLT (Figure 4).

It needs to be mentioned that a large appearance of inward current (i.e., *I*_Na(P)_) following such PT stimulation clearly emerged during the presence of Tef or DLT, while a rather small transient current following the same PT stimuli was observed during the control conditions (i.e., neither Tef nor DLT was present). The larger magnitude of inward current immediately following PT stimuli by adding DLT was noted as compared to that by Tef. Furthermore, the exposure to Tef markedly rendered the inactivation time course of *I*_Na(T)_ during rapid membrane depolarization to become slowed, whereas DLT itself had minimal interference with the inactivation time constant of the current. However, the DLT existence, a progressive elevation of *I*_Na(L)_ and *I*_Na(Tail)_ during a train of repetitive depolarizations; moreover, it induced a larger tail *I*_Na_ following repetitive depolarizations. The experimental results can be interpreted to mean that, upon continued exposure to DLT, the *I*_Na_ deactivation elicited during PT stimuli could be apparently incomplete, thus leading to rate-dependent ‘accumulation’ of the Na_V_ channel activated state. The slowed inactivation caused by the exposure to Tef thus reflects that the barrier for going from the open to the inactivated state of the Na_V_ channel tends to be higher during its presence. The α-cyano-3-phenoxybenzyl group present in the DLT molecule tends to be a notable structure required preferentially for open/resting state of the channel.

Earlier reports have demonstrated the effectiveness of DLT either in inducing the raise in Ca^2+^ transient or exert anti-neoplastic actions in different types of neoplastic cells, including liver, oral, and prostate cancer cells, and Jurkat-J6 cell cells [43,44,45,46,47,48]. The functional expression of Na_V_ channels has also been reported in different neoplastic cells, including prostate cancer and glioma cells [45,49,50,51,52]. As such, whether DLT-mediated modifications on *I*_Na_ presented herein can be responsible for the actions of DLT or other structurally similar pyrethroids on intracellular Ca^2+^ or aberrant growth in neoplastic cells [44] is worth pursuing further.

Earlier reports have demonstrated the effectiveness of pyrethroids (e.g., DLT, esfenvalerate, or permethrin) in increasing long-term potentiation recorded in CA1 hippocampal region [53,54,55,56]. Indeed, a brief period of high-frequency electrical activity applied artificially to a neuronal pathway is expected to enhance the strength of synapses for various periods of time, which is called long-term potentiation. However, it needs to be stressed that with cell exposure to either Tef or DLT, following 1-s PT stimulation from −80 to −10 mV, a large inward current (i.e., *I*_Na(P)_) with slowly decaying process was considerably observed (Figure 4A,B). Under such scenario, the observed induction of long-term potentiation (i.e., facilitation of synaptic transmitter [e.g., glutamate] release) evoked during high-frequency stimulation could have been seriously disturbed or even overestimated by the present findings showing a large recovery time course of *I*_Na_ emerging following PT stimulation in situations where cells present in tissue preparations were exposed to either Tef or DLT.

Previous studies have demonstrated that pyrethroids could affect transepithelial ion transport in the external layers of the skin and the further addition of amiloride could regulate pyrethroids-mediated change in such transepithelial ion transport [16,57]. In this study, the subsequent addition of amiloride can attenuate DLT-induced increase in *I*_Na(L)_ measured from GH_3_ cells; however, further application of chlorotoxin (ChloroTx) had no effect on DLT- or Tef-stimulated *I*_Na_. Moreover, further addition of ChloroTx failed to modify changes in DLT- or Tef-stimulated *I*_Na_ during PT timulation; however, subsequent application of either Dapa or amiloride could significantly attenuate DLT and Tef-stimulated *I*_Na_ by the same stimulation protocol. Therefore, the amiloride-mediated effect on the modifications by pyrethroids of ion transport through rabbit skin is likely associated with its direct inhibitory action on *I*_Na(L)_.

The ability of pyrethroids (e.g., DLT) to augment Cl^−^ currents has been previously demonstrated [14,16]. In our study, the subsequent addition of ChloroTx, an inhibitor of Cl^−^ currents, failed to modify DLT-stimulated *I*_Na(T)_ or *I*_Na(L)_ in GH_3_ cells (Figure 2). However, the further application of dapagliflozin (Dapa) or amiloride can effectively attenuate DLT-activated *I*_Na(L)_ or *I*_Na(P)_. Dapa was recently demonstrated to ameliorate Tef-augmented Hys_(V)_ strength of *I*_Na(P)_ activated by double V_ramp_ [33]. Therefore, DLT-mediated stimulation of *I*_Na(T)_, *I*_Na(L)_, and *I*_Na(P)_ demonstrated herein is unlikely to be attributed to its activation of Cl^−^ current.

Work in our laboratory has demonstrated the non-equilibrium Hys_(V)_ behavior of *I*_Na(P)_ activated by the upright isosceles-triangular V_ramp_ [33]. The results indicated that there was a striking voltage dependence of such V_ramp_-evoked *I*_Na(P)_ [29,33]. The experimental data also showed two types of Hys_(V)_ loops (i.e., a high-threshold counterclockwise followed by a low-threshold clockwise loop) with a figure-of-eight (i.e., ∞) configuration, which is reminiscent of the dynamics of the Lorenz-like motion [58]. Alternatively, with GH_3_-cell exposure to Tef or DLT, the Hys_(V)_ motion of *I*_Na(P)_ activated by the upsloping (ascending) and downsloping (descending) ends of such double V_ramp_ as a function of time was noticed to move in both counterclockwise and clockwise directions (Figure 7A). In particular, one activated during the ascending limb of double V_ramp_ is called a high-threshold counterclockwise loop with a peak of around −10 mV, while the other evoked by the descending limb of V_ramp_ is a low-threshold clockwise loop with a peak falling at around −70 mV [33]. Moreover, as compared with the effect of DLT on Hys_(V)_’s strength of *I*_Na(P)_, the exposure to Tef could augment Hys_(V)_’s strength at low-threshold loop to a greater extent than that observed at high-threshold loop. However, with continued presence of either Tef or DLT, the further addition of Dapa could attenuate their stimulation of Hys_(V)_ strength in GH_3_ cells. Thus, the presence of Tef could slow the inactivation time course of *I*_Na(T)_ activated by rapid step depolarization as well as augment magnitude of *I*_Na(P)_’s low-threshold loop of Hys_(V)_ responding to double V_ramp_. Conversely, as cells were exposed to DLT, the *I*_Na(T)_ inactivation time course during step depolarization was found to remain unchanged, and the increased strength of low-threshold Hys_(V)_ loop during double V_ramp_ was relatively smaller in its presence. It is therefore plausible to assume that the low-threshold loop of *I*_Na(P)_’s Hys_(V)_ activated during the downsloping end of double V_ramp_ could be closely linked to the extent of the inactivation time course of *I*_Na(T)_.

## 4. Conclusions

The modifications by DLT and Tef on the magnitude, gating kinetics, frequency dependence, and Hys_(V)_ strength of *I*_Na_ in electrically excitable cells are noticeably different. The variable actions of pyrethroids presented here would be of clinical, pharmacological, and toxicological relevance [3].

## 5. Materials and Methods

### 5.1. Chemicals, Drugs, Reagents, and Solution Used in This Work

Deltamethrin (DLT, decamethrin, C_22_H_19_Br_2_NO_3_, IUPAC name: [(S)-cyano-(3-phenoxyphenyl)methyl](1R,3R)-3-(2,2-dibromoethenyl)-2,2-dimethylcyclopropane-1-carboxylate, (S)-α-cyano-3-phenoxybenzyl-cis-(1R,3R)-3(2,2-dibromovinyl)(2,2-dimethyl-cyclopropane-carboxylate) was acquired from MedChemExpress (Asia Biomed Inc., Taipei, Taiwan), dapagliflozin (Dapa, Foxiga^®^) was from Cayman (Ann Arbo, MI), while amiloride, tetraethylammonium chloride (TEA), tetrodotoxin (TTX), and tefluthrin (Tef) were from Sigma-Aldrich (Genechain, Kaohsiung, Taiwan). Chlorotoxin was a kind gift from Professor Dr. Woei-Jer Chuang (Department of Biochemistry, National Cheng Kung University Medical College, Tainan, Taiwan). Because of a highly nonpolar nature of low water solubility (Laskowski, 2002), the stock solution of DLT (10 mM) was prepared by dissolving it in dimethylsulfoxide (DMSO), and it was wrapped in aluminum foil and then kept under −20 °C for long-term storage. Unless specified otherwise, growth media (e.g., Ham’s F-12 medium), fetal or horse bovine serum, trypsin/EDTA, and L-glutamine were mostly acquired from HyClone^TM^ (Thermo Fisher, Kaohsiung, Taiwan), while other chemicals or reagents were from Sigma-Aldrich or Merck (Genechain), and they were of laboratory grade and taken from standard sources.

The standard extracellular solution (i.e., normal Tyrode’s solution) used in this study had the ionic compositions containing (in mM): NaCl 136.5, CaCl_2_ 1.8, KCl 5.4, MgCl_2_ 0.53, glucose 5.5, HEPES 5.5, and the solution pH was titrated to 7.4 by adding NaOH. The composition of Ca^2+^-free Tyrode’s solution used for the measurement of *I*_Na_ (e.g., *I*_Na(T)_, *I*_Na(L)_, *I*_Na(P)_, and *I*_Na(Tail)_) was the same as normal Tyrode’s solution in which CaCl_2_ was removed. For the experiments on recording *I*_Na_, the electrode used was filled up with the internal pipette solution containing (in mM): Cs-aspartate 130, CsCl 20, KH_2_PO_4_ 1. MgCl_2_, Na_2_ATP 3, Na_2_GTP 0.1, and HEPES 5, and the pH was then adjusted to 7.2 with CsOH. The twice-distilled water used for the experiments was deionized with a Milli-Q ion exchange and activated carbon cartridge treatment system (Merck, Tainan, Taiwan).

### 5.2. Cell Preparation

Clonal pituitary (GH_3_) somatolactotrophs, originally acquired from the Bioresources Collection and Research Center ([BCRC-60015], http://catalog.bcrc.firdi.org.tw/BcrcContent?bid=60015) (access on 19 September 2022), Hsinchu, Taiwan), were revived and cultured in Ham’s F-12 growth medium, which was supplemented with 15% heat-inactivated horse serum (*v*/*v*), 2.5 % fetal calf serum (*v*/*v*), and 2 mM L-glutamine. They were commonly incubated at 37 °C in monolayer cultures in 50-mL plastic culture flasks in a humidified environment of 5% CO_2_/95% air. It was confirmed that this cell line can continually secrete prolactin. We carried out electrical recordings 5 or 6 days after cells underwent subculture (60–70% confluence).

### 5.3. Electrophysiological Measurements (Patch-Clamp Current Recordings)

In the few hours before the experiments, GH_3_ cells were detached from culture dishes with a 1% trypsin/EDTA solution, and a few drops of cell suspension (~10^6^/mL) was rapidly placed in a custom-built chamber mounted on the stage of a DM-IL inverted phase-contrast microscope (Leica; Major Instruments, Kaohsiung, Taiwan). We bathed cells at room temperature (20–25 °C) in the extracellular solution (i.e., normal Tyrode’s solution), the ionic compositions of which are described above. Before each experiment, cells were allowed to settle on the chamber’s bottom. The recording pipettes were pulled from Kimax^®^-51 borosilicate glass tube (#DWK34500-99; Kimble^®^, Merck, Tainan, Taiwan) and they were then polished to reach the resistances ranging between 3 and 5 MΩ. During each measurement, the electrode was mounted in an air-tight holder, which had a suction port on the side, and a silver-chloride wire was used to make good contact with the internal pipette solution. We recorded varying types of ionic currents (e.g., *I*_Na_) with the whole-cell mode of a modified patch-clamp technique by using an RK-400 patch amplifier (Bio-Logic, Claix, France), as dealt with in our previous works [26,36,59]. All recordings were conducted inside a noise-proof Faraday cage. The junction potentials that commonly develop when the compositions of the pipette internal solution are different from those in the bath were zeroed shortly before giga-Ω formation was made, and the whole-cell data were corrected. As pulse train (PT) stimulation was applied to the tested cell, we used an Astro-Med Grass S88X dual output pulse stimulator (Grass; KYS Technology, Tainan, Taiwan).

### 5.4. Data Recordings and Processing

Throughout the recording period, the signal output (i.e., potential and current traces) was monitored and digitized online at 10 kH or more in an ASUS ExpertBook laptop computer (Yuan-Dai, Tainan, Taiwan). For analog-to-digital (A/D) and digital-to-analog (D/A) conversion, a Digidata^®^ 1550B converter equipped with the computer was controlled by pCLAMP^TM^ 10.6 program run under Microsoft Windows 7 (Redmond, WA, USA). Current signals were low-pass filtered at 2 kHz by using a FL-4 four-pole Bessel filter (Dagan, Minneapolis, MN, USA). The voltage-clamp protocols with manifold rectangular or ramp waveforms were designed, and they were then given to the examined cell through D/A conversion.

### 5.5. Data Analyses for Whole-Cell Ionic Currents

To establish concentration-dependent stimulation of DLT on the amplitude of *I*_Na(T)_ or *I*_Na(L)_, we bathed GH_3_ cells in Ca^2+^-free Tyrode’s solution which contained 10 mM tetraethylammonium chloride (TEA). During the recording period, we voltage-clamped each cell at −80 mV, and a brief step depolarization to −10 mV for a 20 ms at a rate 0.2 Hz was applied to evoke *I*_Na_. The *I*_Na(T)_ magnitude was measured as the peak amplitude of *I*_Na_ at the beginning of depolarizing pulse was subtracted from the sustained *I*_Na_ (i.e., *I*_Na(L)_), while the *I*_Na(L)_ magnitude was measured at the end of 20-ms depolarizing pulse in situations where different DLT concentrations were cumulatively given (as indicated in the right side of Figure 1A). The total amplitude of *I*_Na_ (*I*_Na(Tot)_) taken from each step depolarization is equal to *I*_Na(T)_ plus *I*_Na(L)_. The amplitude of *I*_Na(L)_ obtained during the presence of DLT at a concentration of 100 µM was considered as 100% and we then compared current magnitudes (i.e., *I*_Na(T)_ and *I*_Na(L)_) during cell exposure to varying concentrations of DLT. The concentration-dependent stimulation by DLT of *I*_Na(T)_ or *I*_Na(L)_ observed in GH_3_ cells was determined by fitting experimental data set to a modified Hill function [10,25], which can be given as follows.

$$\mathrm{percentage}\;\mathrm{increase}\;\left(\%\right)=\;\langle E_{\max}\times {\left[\mathrm{DLT}\right]}^{n_{H}}\rangle /\langle {\mathrm{EC}}_{50}^{n_{H}}+{\left[\mathrm{DLT}\right]}^{n_{H}}\rangle$$

In this equation, [DLT] = the deltamethrin (DLT) concentration given; n_H_ = the Hill coefficient (i.e., coefficient for cooperativity); EC_50_ = the concentration required for a 50% stimulation of *I*_Na(T)_ or *I*_Na(L)_ amplitude activated in response to short depolarizing step from −80 to −10 mV; and E_max_ = maximal stimulation of *I*_Na(T)_ or *I*_Na(L)_ produced by the DLT presence.

### 5.6. Curve-Fitting Approximations and Statistical Analyses Used in This Work

To determine the model parameters, linear or nonlinear curve-fitting to the experimental data set presently obtained was optimally fitted with least-squares minimization procedure by using manifold analytical tools, such as the Microsoft “Solver” built in Excel^®^ 2022 (Microsoft) and OriginPro^®^ 2022 program (OriginLab^®^; Scientific Formosa, Kaoshiung, Taiwan). The experimental results are presented as the mean ± standard error of the mean (SEM). The size of independent observations (*n*) is indicated in cell numbers collected during the measurements. The data distribution obtained presently was found to satisfy the tests for normality. Paired or unpaired *t*-tests were used for comparison between the two different groups; however, for comparison among more than two groups, we carried out analysis of variance (for one- or two-way ANOVA) with or without repeated measure followed by a post hoc Fisher’s least-significance difference test for multiple-range comparisons. A statistical significance (indicated with *, **, ^+^, or ^++^ in the figures) was considered when *p* < 0.01 or < 0.05.

## Figures and Tables

**Table 1:** Effects of DLT, DLT plus dapagliflozin (Dapa, 10 mM), and DLT plus amiloride (Ami, 10 mM) on either the decaying time constant of I_Na(T)_ during pulse train (PT) stimulation (i.e., cumulative inhibition of I_Na(T)_ during rapid depolarizing stimuli) or the rising time constant of I_Na(L)_ during the same PT stimulation, as well as the time constant of I_Na(Tail)_ recovery evoked following PT stimulation. All values are mean ± SEM.

	Control	DLT (10 mM)	DLT (10 mM) Plus Dapa (10 mM)	DLT (10 mM) Plus Ami (10 mM)	Cell Number (*n*)
Decaying time constant of *I*_Na(T)_	22.1 ± 2.8 ms	56.4 ± 3.9 * ms	29.6 ± 6.1 * ms	30.9 ± 6.5 * ms	8
Rising time constant of *I*_Na(L)_	(-)	87.4 ± 4.6 ms	19.1 ± 6.1 ** ms	21.1 ± 6.5 ** ms	8
Recovery time constant of *I*_Na(Tail)_	25 ± 3 ms	1.23 ± 0.19 ^+^ s	0.56 ± 0.04 ** s	0.58 ± 0.05 ** s	8

* Significantly different from controls (*p* < 0.05), ** significantly different from DLT (10 mM) alone groups (*p* < 0.05), and ^+^ significantly different from controls (*p* < 0.01). (-) shown in Table 1 indicates that the time constant of *I*_Na(L)_ during PT stimulation decayed in an exponential manner.

## Data Availability

The original data is available upon reasonable request to the corresponding author.

## Abbreviations

DLT	deltamethrin
ChloroTx	chlorotoxin
Dapa	dapagliflozin
EC_50_	concentration required for half-maximal stimulation
Hys_(V)_	voltage-dependent hysteresis
*I-V* relationship	current versus voltage relationship
*I*_Na_	voltage-gated Na^+^ current
*I*_Na(L)_	late Na^+^ current
*I*_Na(P)_	persistent Na^+^ current
*I*_Na(Tot)_	total Na^+^ current (i.e., *I*_Na(T)_ plus *I*_Na(L)_)
*I*_Na(Tail)_	tail Na^+^ current
Na_V_ channel	voltage-gated Na^+^ channel
PT stimulation	pulse train stimulation
Tef	tefluthrin
V_ramp_	ramp voltage
